# HIV treatment outcomes following antiretroviral therapy initiation and monitoring: A workplace program in Papua, Indonesia

**DOI:** 10.1371/journal.pone.0212432

**Published:** 2019-02-25

**Authors:** Yuriko Limmade, Liony Fransisca, Rodrigo Rodriguez-Fernandez, Michael J. Bangs, Camilla Rothe

**Affiliations:** 1 Institute of Tropical Medicine and International Health, Charité- Universitätsmedizin Berlin, Germany; 2 Kuala Kencana Clinic, PT Freeport Indonesia/International SOS, Papua, Indonesia; 3 Non-Communicable Diseases, International SOS, London, United Kingdom; 4 NCD Asia Pacific Alliance, Tokyo, Japan; 5 Public Health & Malaria Control Department, PT Freeport Indonesia/International SOS, Papua, Indonesia; 6 Division of Infectious Diseases and Tropical Medicine, University Hospital, LMU Munich, Germany; The Ohio State University, UNITED STATES

## Abstract

**Background:**

Papua Province, Indonesia is experiencing an on-going epidemic of Human Immunodeficiency Virus (HIV) infection, with an estimated 9-fold greater prevalence than the overall national rate. This study reviewed the treatment outcomes of an HIV-infected cohort on Antiretroviral Therapy (ART) and the predictors in terms of immunological recovery and virological response.

**Methods:**

ART-naïve individuals in a workplace HIV program in southern Papua were retrospectively analyzed. Patients were assessed at 6, 12 and 36 months after ART initiation for treatment outcomes, and risk factors for virological suppression (viral load (VL) <1,000 copies/ml), poor immune response (CD4 <200 cells/mm^3^) and immunological failure (CD4 <100 cells/ mm^3^) after at least 6 months on ART, using a longitudinal Generalized Estimating Equations multivariate model.

**Results:**

Assessment of 105 patients were included in the final analysis with a median age of 34 years, 88% male, median baseline CD4 236 cells/ mm^3^, and VL 179,000 copies/ml. There were 74, 73, and 39 patients at 6, 12, and 36 months follow-up, respectively, with 5 deaths over the entire period. For the three observation periods, 68, 80, and 75% of patents achieved virological suppression, poor immune responders decreased from 15, 16 to 10%, whilst 15, 16, 10% met the immunological failure criteria, respectively. Using multivariate analysis, the independent predictor for viral suppression at 12 and 36 months was ≥1 log decrease in VL at 6 months (OR 19.25, p<0.001). Higher baseline CD4 was significantly correlated with better immunological outcomes, and lower likelihood of experiencing immunological failure (p <0.001).

**Conclusion:**

Virological response at six months after beginning ART is the strongest predictor of viral suppression at 12 and 36 months, and may help in identifying patients needing additional adherence therapy support. Higher baseline CD4 positively affects the immunological outcomes of patients. The findings indicate HIV control programs should prioritize the availability of VL testing and begin ART regardless of CD4 counts in infected patients.

## Introduction

Virological monitoring is the preferred method to monitor people living with HIV/ AIDS (PLWHA) on Antiretroviral Therapy (ART) [1]. Viral load (VL) monitoring is used to evaluate treatment progress, detect treatment failure, and assess transmission risk [1–3]. However, like many resource-limited settings, routine VL monitoring is not readily available in Papua, a remote area of Indonesia [4,5].

Home to the world’s fourth largest population of more than 260 million people [6], the overall prevalence of HIV in Indonesia is considered relatively low (97.8 per 100,000), with epidemic-level transmission occurring predominantly in Papua Province [7,8]. Papua Province has high HIV prevalence (872.6 per 100,000), nearly nine times greater than the nation-wide rate [8–10]. Mimika District, a large and sparsely populated area in southern Papua Province, has an estimated HIV prevalence of 1,337 per 100,000; more than thirteen times higher than the national rate [11]. The risk of HIV transmission in Indonesia is predominately through high-risk heterosexual behaviors [8].

With good adherence, antiretroviral drugs (ARVs) improve the overall health status, survival and quality of life of PLWHA [12–17]. Beginning in 2016, the HIV treatment policy in Mimika targets all infected individuals with ART coverage, adhering to the revised WHO guidelines [1,11]. Treatment coverage increased from 54% in 2013 to 83% in 2015, while reverting back to 54% in 2016, the downturn possibly related to the expanded ARV eligibility policy. In spite of increased ART coverage, the annual HIV-related mortality in the district has increased over a 4-year period from 6% to 11% beginning in 2013 [4, 11]. The reason for this apparent increase in mortality is unclear. Possible reasons include poor adherence to ART and treatment failure, but also improved surveillance during this period.

PT Freeport Indonesia (PTFI), an affiliate operation of an international gold and copper mining company, has operated in the Mimika District since the 1970s. Through its medical service provider, it began an HIV awareness campaign in the mid-1990s and an organized HIV treatment program in 2007, in parallel and partnership with the national HIV program at the district level. The company provides free-of-charge HIV testing and counseling, as well as care and treatment services, with the support of the national program and district health authorities for providing the ARV drug supplies. At the time of this study, all HIV-infected patients under the company’s clinical care were offered to start ART following the most current WHO recommendations [1].

Studies have shown a large proportion of treatment failures attributed to patient non-adherence [1,18,19]. This is aggravated by the lack of VL testing which leads to the under diagnosis of treatment failure [19–23]. The current Indonesian National Guidelines on ART (2014) places less emphasis on virological monitoring for treatment guidance, unless testing is available or treatment failure is suspected [9]. In most areas of Indonesia, patient monitoring after beginning ART remains limited to CD4-count measurements.

Currently, VL testing is not performed on a regular basis in the city of Timika (Mimika District capital), and is rarely accessible to the general public. However, PTFI health services routinely performs virologic monitoring amongst a sub-population (mine employees), in addition to the standard laboratory work-ups recommended in the 2014 guidelines.

This study is designed to assess treatment outcomes and predictors among HIV positive adults initiating first-line ART under a workplace HIV treatment program. Until now, a detailed assessment of treatment outcomes in this particular group remained incomplete.

## Methods

### Study design and eligibility criteria

We performed a retrospective cohort study of ART-naïve patients (employees and their immediate beneficiaries residing at site), ≥ 18 years-of-age, who began their first-line ART regimen at the mine-managed health care facilities between January 2008 and October 2015. Treatment outcomes were assessed at 6, 12, and 36-month time points, with repeated CD4 and VL level measurements. All patients were followed until their last visit, death, or end of the study period (36 months after beginning ART). Individuals without recorded baseline CD4 and VL measures, and at least one subsequent six-monthly CD4 and VL measures were excluded. Pregnant women at ART initiation or during the follow-up period were either excluded or censored, respectively. The reasoning behind removing pregnancy is that hemodilution, particularly during late stage pregnancy, might interfere with the interpretation of absolute CD4 cell counts [24]. For patients who were lost to follow-up during the observation period, only available laboratory results were included in the analysis.

### Variables

Treatment response was considered favorable in cases with viral suppression (VL <1,000 copies/ml) after a minimum of 6 months on ART. Patients failing to achieve a CD4 cell count of 200 cells/mm^3^ or above were categorized as “poor immune responders”. Immunological failure was defined as persistently low CD4 count below 100 cells/ mm^3^.

### Data collection

Routine data were accessed through the on-site electronic database medical record system. Data collected included basic demographic parameters such as age, sex, ethnicity, ART regimen, baseline CD4 counts and VL levels immediately prior to ART. Evaluations of VL level were based on a single measurement. To protect confidentiality, all patient identifiers were removed from the database before analysis.

### Analysis

Patients were grouped according to their baseline CD4 count and VL level at the start of ART and other demographic variables. Categorical variables were summarized as frequencies and percentages while numerical variables with non-normal distributions were summarized as median and interquartile range (IQR).

We evaluated three endpoints: proportions of patients achieving viral suppression, failing to achieve a minimal CD4 count of 200 cells/mm^3^, and experiencing immunological failure. Univariate analysis was conducted using chi-square (χ^2^) test. Predictors with a p-value <0.1, were placed in a longitudinal Generalized Estimating Equations (GEE) multivariate model to fit a repeated measures logistic regression. An independence correlation structure was used for the assumption that each repeated endpoint measurement is independent from one other. A 2-sided, p-value <0.05 was deemed statistically significant. All statistical calculations were conducted using STATA version 13.1 (StataCorp, College Station, TX).

### Ethical approval

The Medical and Health Research Ethics Committee (MHREC), Faculty of Medicine, Gadjah Mada University, Indonesia granted ethical approval (KE/FK/1302/EC/2016), followed by final study approval from PTFI. This study used only secondary data; i.e., laboratory records and information from routine clinical records, therefore patients were not required to provide written consent for study participation.

## Results

**Fig 1:**
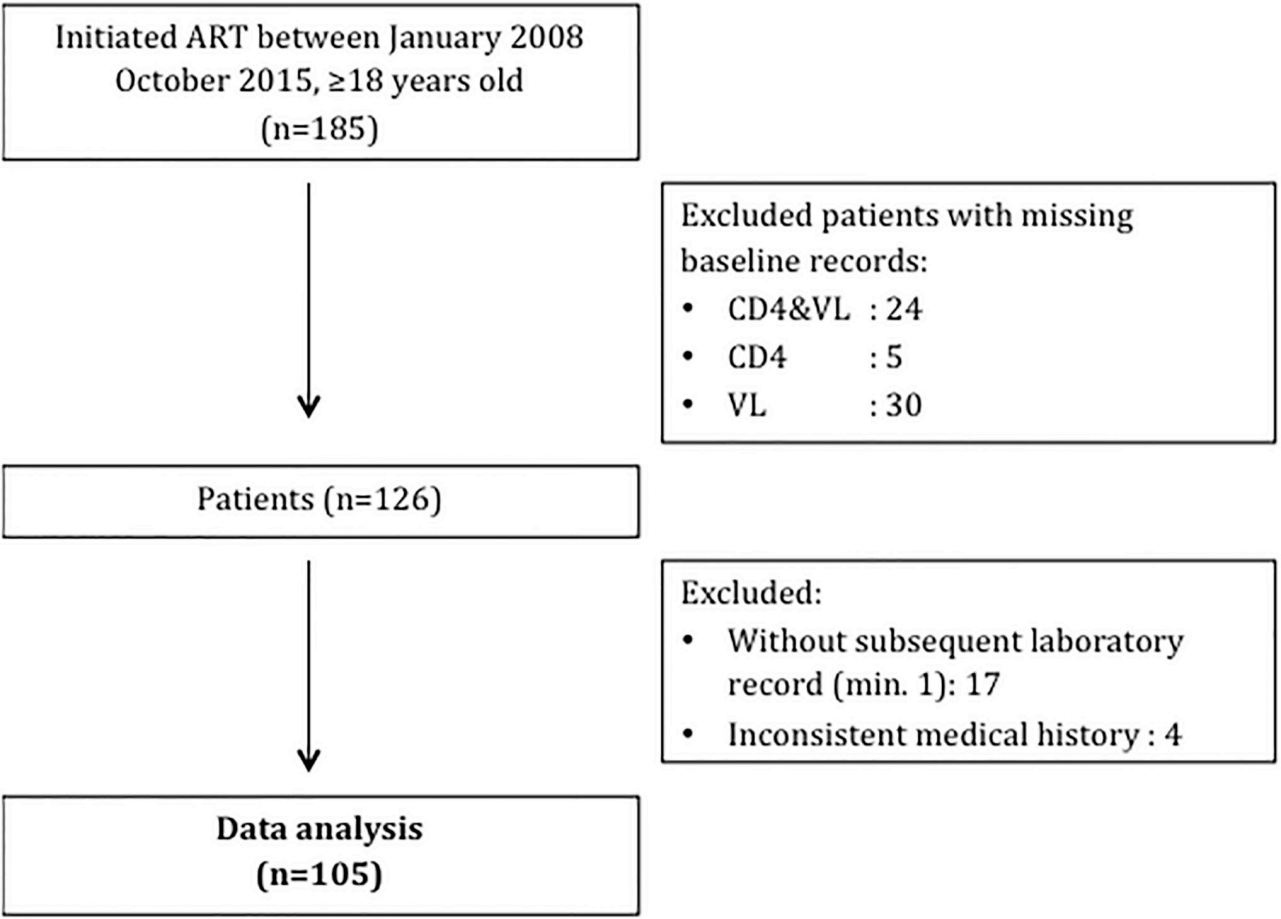
HIV treatment outcomes study tree.

Between January 2008 and October 2015, 185 HIV positive patients initiated their first ART regimen at the participating health care centers. Of these, 76 patients were excluded, as they had one or more exclusion criteria. Four further patients were excluded due to inconsistent medical records where date of first ART initiation could not be determined (Fig 1).

Baseline characteristics of the study population are provided in Table 1. Of the 105 patients enrolled, 88% were male, with a median age of 34 years [interquartile range (IQR) 29–38] at ART initiation. The majority (83%) of patients were of indigenous Papuan ethnicity. Non-Papuan patients (17% of study cohort) represented other Indonesian national ethnicities originating from outside of Papua. The median baseline CD4 cell count was 236 cells/ mm^3^ (IQR, 115–306), and median baseline plasma VL level was 179,000 copies/ml (IQR, 75,000–655,000).

**Table 1:** Baseline demographic and HIV infection characteristics of study population.

Variable	Number (%)	Median	IQR
Sex (n = 105)			
Male	92 (88)		
Age at ART initiation, years (n = 105)		34	29–38
18–29	31 (30)		
30–39	53 (50)		
40–49	20 (19)		
≥50	1 (1)		
Ethnic group (n = 105)			
Papuan 7-tribes*	29 (28)		
Papuan**	58 (55)		
Non-Papuan	18 (17)		
CD4 count at ART initiation, cells/ mm3 (n = 105)		236	115–306
Low, ≤100	22 (21)		
Medium, 101–350	61 (58)		
High, >350	22 (21)		
VL level at ART initiation, copies/ml (n = 105)		179,000	75,000–655,000
Low, <10,000 (4log_10_)	7 (7)		
Medium, 10,000–100,000 (4log_10_-5log_10_)	29 (27)		
High, >100,000 (>5log_10_)	69 (66)		

*Native ethnic groups of Mimika & adjoining Districts; Amungme, Kamoro, Dani, Moni, Mee/Ekari, Damal and Nduga

** Other Papuan ethnic groups outside of the 7 tribes

At the end of the observation period, with 36 months of follow-up after beginning ART, 4% of study subjects had died (n = 5), and 13% were no longer active in the system (n = 16 lost to retrospective follow-up, e.g., either not attending clinic or no longer working in the company). Two patients (1.6%) had switched to the second-line regimen (n = 2). Laboratory findings following the exclusionary event were not included in the final analysis.

### Immunological recovery and virological response

**Fig 2:**
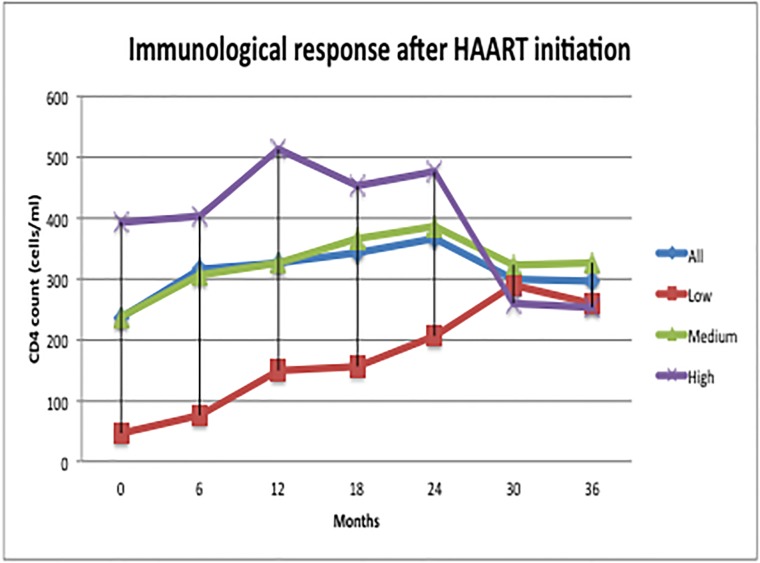
Immunological recovery after ART initiation stratified by baseline CD4 groups.

Temporal treatment response and outcome measures by baseline CD4 groups are presented in Table 2. The average median CD4 count increased from 237 cells/ mm^3^ (IQR, 115–322) at baseline, to 303 cells/ mm^3^ (IQR, 221–432) at the end of 36 months. The largest increment in CD4 count was seen in the ‘Low’ baseline CD4 group, compared to ‘Medium’ and ‘High’ groups, from a median of 46 cells/ mm^3^ (IQR, 20–76), 236 cells/ mm^3^ (IQR, 168–281), and 396 cells/ mm^3^ (IQR, 367–436) at baseline, to 266 (IQR, 203–400), 325 (IQR, 235–453), and 253 cells/ mm^3^ (IQR, 128–406) at 36 months, respectively (Fig 2).

**Table 2:** Temporal HIV treatment outcomes separated by baseline CD4 groups.

Treatment outcome, time points (months)	ALL, n (%)	Baseline CD4 group*			P value
		Low, n (%)	Medium, n (%)	High, n (%)	
**Poor immune responder, CD4<200 cells/mm3**					
**6**	21/74 (28)	12/17 (71)	8/41 (20)	1/16 (6)	**<0.001**
**12**	23/73 (32)	11/17 (65)	10/42 (24)	2/14 (14)	**0.003**
**36**	7/39 (18)	2/9 (22)	4/26 (15)	1/4 (25)	0.834
**Immunological failure, CD4<100 cells/mm3**					
**6**	11/74(15)	8/17 (47)	3/41 (7)	0/16 (0)	**<0.001**
**12**	12/73 (16)	6/17 (35)	4/42 (10)	2/14 (16)	0.052
**36**	4/39 (10)	1/9 (11)	2/26 (8)	1/4 (25)	0.566
Viral suppression LMIC**, VL<1000 copies/ml					
**6**	43/63 (68)	12/17 (71)	25/36 (69)	6/10 (60)	0.827
**12**	48/60 (80)	14/18 (78)	25/31 (81)	9/11 (82)	0.958
**36**	21/28 (75)	5/8 (63)	15/17 (88)	1/3 (33)	0.081
**Median CD4 count, IQR, cells/mm3**					
**Baseline (n = 105)**	237, 115–322	46, 20–76	236, 168–281	396, 367–436	
**6 Month (n = 74)**	286, 184–371	125, 41–207	283, 215–358	401, 310–608	
**12 Month (n = 73)**	301, 180–468	142, 67–235	326, 220–461	512, 380–532	
**36 Month (n = 39)**	303, 221–432	266, 203–400	325, 235–453	253, 128–406	
Median VL level, IQR copies/ml***					
**Baseline (n = 105)**	179,000, 75,000–655,000	387,000, 158,000–1,310,000	154,000, 65,750–472,000	142,000, 65,800–638,000	
**6 Month (n = 74)**	270, <20–4,540	728, 306–1,110	54.5, <20–3,815	56, <20–51,800	
**12 Month (n = 73)**	42.5, <20–455	159, 23–731	29, <20–418	<20, <20–384	
**36 Month (n = 39)**	21.5, <20–6,667	567, <20–94,750	<20, <20–39	205,000, <20–2,040,000	

Note

* Baseline (ART initiation) CD4 group: LOW ≤100, MEDIUM 101–350, HIGH >350cells/mm3

**Low-middle income countries (LMIC),

***Limit of detection assay used<20copies/ml

Significance set at p< 0.05.

Of the patients that were followed and had complete laboratory assays entered for 6, 12, and 36 months (n = 74, 73, 39, respectively), 28% (21 of 74), 32% (23 of 73) and 18% (7 of 39) showed poor immunological response marked by CD4 count below 200 cells/mm^3^, respectively. Of these, 15%, 16%, and 10% of patients, respectively, met the criteria for immunological failure as persistently low CD4 level <100 cells/ mm^3^.

At 6 months following ART initiation, 68% (n = 74) of the treated patients achieved viral suppression marked by VL level <1,000 copies/ml, increasing to 80% (n = 73) at 12 months, and 75% (n = 39) at 36 months. Median plasma VL declined from 175,000 copies/ml (IQR, 73,100–696,000) at baseline to 22 copies/ml at 36 months, much lower than the defined limit of detection for suppression threshold criteria in LMIC [25].

### Predictors of immunological recovery and viral suppression

Univariate analyses revealed that neither sex nor baseline VL level affected treatment response. Multivariate analyses revealed that the single predictor of favorable virological outcome at 12 and 36 months was a one-log decrease in VL level at 6 months post-ART initiation (OR = 19.25, 95% 3.91–94.78, p <0.001). Patients of non-Papuan ethnicity have a higher likelihood (OR = 10.9, 95% CI 2.5–47.46, p = 0.001) of achieving viral suppression at 6, 12, and 36-month time points, compared to those of Papuan ethnicity (Table 3).

**Table 3:** Logistic regression (GEE) analysis of factors influencing HIV treatment outcomes at 6, 12 and 36 months using multivariate analysis comparisons across low, medium and high groups.

Parameter	Viral suppression LMIC, VL <1000 copies/ml		Poor immune responder, CD4 <200 cells/mm3		Immunological failure, CD4 <100 cells/mm3	
	Adj. OR (95% CI)	P Value	Adj. OR (95% CI)	P Value	Adj. OR (95% CI)	P Value
**Ethnicity**						
Papuan 7-tribes	1		1		1	
Papuan	1.32 (0.49–3.54)	0.59	0.55 (0.21–1.40)	0.21	0.57 (0.19–1.71)	0.31
Non-Papuan	**10.90 (2.50–47.46)**	**0.001**	**0.25 (0.07–0.87)**	**0.03**	**0.08 (0.01–0.60)**	**0.014**
**Age group**			-	-		
18–29	1		-	-	1	
30–39	2.31 (0.44–12.07)	0.32	-	-	**0.22 (0.05–0.87)**	**0.03**
40–49	3,88 (0.48–31.33)	0.2	-	-	0.31 (0.07–1.43)	0.13
>50	-	-	-	-	-	-
**Baseline CD4 group**						
Low, ≤100	1		1		1	
Medium, >100–350	2.78 (0.71–10.86)	0.14	**0.16 (0.06–0.42)**	**<0.001**	**0.08 (0.02–0.29)**	**<0.001**
High, >350	2.83 (0.50–16.13)	0.24	**0.12 (0.03–0.43)**	**0.001**	**0.18 (0.03–0.95)**	**0.044**
**1 log decrease in VL at 6 months**						
No	1		-	-	-	-
Yes	19.25 (3.91–94.78)*	**<0.001**	-	-	-	-

Note:

* Viral suppression outcomes at 12 and 36 months.

Significance set at p <0.05.

Patients with higher baseline CD4 counts were significantly less likely to be poor immune responders; ‘medium’ (OR = 0.16, 95%CI 0.06–0.42, p <0.001) and ‘high’ CD4 counts (OR = 0.12, 95%CI 0.04–0.43, p = 0.001); or experiencing immunological failure; ‘medium’ (OR = 0.08, 95%CI 0.02–0.29, p <0.001) and ‘high’ CD4 counts (OR = 0.18, 95%CI 0.03–0.95, p = 0.044); at 6,12 and 36 month time points whilst on ART, than patients in the ‘low’ baseline CD4 group (Table 3). Additionally, patients in the ‘30–39’ age group appear less likely to have immunological failure (OR = 0.22, 95% CI 0.05–0.87, p = 0.03) than those in the ‘18–29’ age group.

Ethnicity was also associated with immunological outcomes at 6, 12, and 36 months. Non-Papuan patients were less likely to be poor immune responders (OR = 0.25, 95% CI 0.07–0.87, p = 0.03) and had a lower likelihood of experiencing immunological failure (OR = 0.08, 95% CI 0.01–0.60, p = 0.014) whilst on ART, when compared to patients of Papuan descent.

## Discussion

Our findings indicate that patients overall showed improvements in both virological and immunological response whilst on ART. Virological suppression was achieved in 80% of our study population at 12 months after ART initiation. This is within the range of 71–84% of patients showing viral suppression after 12 months of ART, reported by the TAHOD study and other HIV programs in LMIC settings [26,27]. It is also in accordance with WHO global fair performance standard; though the more desirable performance target is above 90% [28]. Viral suppression should be the primary outcome for all patients on ART, as survival is dependent on the achievement and maintenance of a suppressed viral load [29].

In our study, we found that the most significant predictor of achieving viral suppression at 12 and 36 months was a 1-log decrease in VL at six months after beginning ART. This finding is concordant with other studies reporting that a decrease in VL at an earlier stage showed a strong association with better virological outcomes at 12–24 months post- ART initiation [30–32]. In our setting there was very limited ART exposure before the study, and hence transmitted primary resistance to first-line therapy is unlikely; indicating poor virological outcome is most likely due to poor ART adherence. In the Belgrade cohort study, survival of patients on ART was solely dependent on the achievement and maintenance of viral suppression, regardless of immune recovery and baseline characteristics [29]. Therefore, virological monitoring should be prioritized, and suboptimal adherence identified and remedied as early as possible to reduce risk of drug resistance and transmission, and provide better virological outcome.

In terms of immunological outcomes, the majority of patients showed improvements in CD4 count recovery over time. As there is no consensus on the definition of successful immunological recovery, we were interested in the minimal CD4 count before opportunistic infections typically become evident in poor immune responders. An absolute CD4 count of less than 200 cells/mm^3^ at 6 months post-ART initiation increases the risk of disease progression and death [15,33]. A collaborative cohort study in the United Kingdom also showed that sub-optimal CD4 increases heightened the risk of death but not new AIDS events [34]. The proportion of patients in our study achieving this threshold increased over time in all baseline CD4 groups, thus lowering their risk of more severe or complicated morbidity and mortality.

A low CD4 count at ART initiation was associated with higher likelihood of being a poor immune responder in our series. This is in concordance with other studies, which reported baseline CD4 counts were positively associated with immunological recovery [29,33]. This supports the most recent WHO guidelines, which advocates the initiation of ART for all HIV infected individuals regardless of CD4 count [1]. Similar to other published studies [14,33,35,36], we have shown that proportionally, immunological recovery was highest in patients who had begun treatment with a ‘low’ (<100 cells/ mm^3^) count at baseline. However, despite showing the highest relative increase in CD4 recovery, patients in the ‘low’ group achieved lower CD4 peaks than those with higher baseline groups. Observation time, wherein an increase in CD4 depends on both the baseline value and amount of time on continuous treatment, might be one possible explanation for our findings [33]. Nonetheless, ART treatment should begin as early as possible, irrespective of baseline values.

Immunological failure was significantly associated with lower initial CD4 counts and age. Other studies [33,37,38] have reported that older age (> 40 years) increases the probability of immunological failure, which might be due, in part, to the reduced thymic function of the patient [39]. However, we did not find a correlation between older age and immunological response (i.e., failure); rather, ‘30–39’ year old patients on ART were less likely to experience immunological failure than those in the ‘18–29’ age group. Other factors that might influence this finding such as marital status, behavior (lifestyle), ART adherence (consistency), and other socio-demographic data [37,40] were unavailable and warrant further investigation.

One important finding in our study was that ethnicity appeared to be a significant predictor of favorable treatment outcomes. Patients of non-Papuan origin (e.g., predominately Javanese and other non-Papuan Indonesians) have a higher likelihood of achieving viral suppression, a lower likelihood of being a poor immune responder, and experiencing immunological failure, compared to patients belonging to the Papuan 7-tribes. There were no significant differences in ART response between the Papuan ethnic groups (7-tribes vs. others). The correlation between therapeutic response and ethnicity alone is not clear. Ethnicity (i.e., genetic factors) itself may not be a causal factor, but could influence immunological response for other reasons. However, we believe that social determinants such as prevailing cultural practice, educational level (e.g., literacy, awareness), as well as disease perceptions and treatment-seeking behavior may play an important role in a patient’s adherence to treatment and thus affecting treatment outcome. We could not find relevant treatment outcome studies in Papua or Papua New Guinea settings, published investigations were mostly related to the epidemiology, social experiences of PLWHA on ART as well as prevention initiatives [41–44]. As Papuans represent the vast majority of detected HIV cases in the workforce (e.g., 83% of followed subjects in this study), while representing around 30% of the total workforce, it deserves a more thorough investigation in order to curb the higher incidence of infection in these more vulnerable at-risk groups.

Interestingly, similar to longitudinal studies conducted in Ethiopia [45,46], we observed a sharp temporal decline in CD4 counts between 24 and 30 months amongst patients starting ART with higher counts at treatment start. However, in both Ethiopian studies, the observation occurred at a later period d into therapy. A possible reason for our findings could be due to the remarkably higher levels of viremia in the ‘High’ group at that specific time point, and also the low number of patients in the ‘High’ group that were seen at specific time points. It has been suggested that the immunological recovery after ART initiation is mainly due to decreasing VL and the redistribution of memory CD4 cells [47–49]. The largest proportion of study patients without follow-up laboratory results were those with higher baseline CD4 levels. As these individuals may presumably have had slower disease progression and thus possibly more likely to have avoided outpatient visits (“walking well” phenomenon), this may have contributed to observed temporal decline.

There are several limitations in this retrospective study. VL level assessments were done based on single laboratory measurements, which might have affected the analysis by not ruling out random viral ‘blips’ (intermittent low-level viremia). The initial number of patients for inclusion was limited, and became smaller after invoking the study exclusion criteria. There was also a high percentage (~52%) of loss to follow-up patients by the end of the 36-month observation, especially in the ‘High’ CD4 group. This may have underestimated the outcome in the ‘High’ group. Health care providers should make a concerted effort to trace the LTFU. Additionally, since this study used secondary data derived from routine medical records, possible issues of data integrity may have had an impact on analyses and conclusions. To maximize the analytic precision, a longitudinal GEE multivariate model allowed use of all available data from each study subject. Advantages of this approach include accounting for correlations between response variables across time in the same individual and the flexibility to use non-normal distributed data without the need of complete datasets [50].

Other points of interest such as patient HIV awareness and perceptions, actual daily adherence to treatment, education level, location of residence (onsite/ off-site), and disclosure of HIV-status to close acquaintances, were not available for analysis. The five deaths observed in our followed cohort had limited data for further analysis. Lastly, because employment at the mine is highly skewed towards male workers, females, in general, were under-represented in the analysis (12% of 105 patient records).

## Conclusions

Our findings demonstrate that VL testing plays a crucial role in predicting favorable virological outcome whilst on ARV treatment, and that baseline CD4 is the most significant predictor of subsequent immune recovery. National HIV control programs at the district level, should strongly consider implementing virological monitoring and provide VL testing for the public to enhance better treatment outcomes. To our knowledge, there are very few, if any, routine virological monitoring programs measuring treatment response outcomes in Indonesia. These findings from a workplace program should be a useful benchmark for other HIV programs in the country for considering virological monitoring of patient prognosis and treatment progress.

## Supporting information

S1 Table Univariate analyses of predictors of HIV treatment outcomes across low, medium and high groups at 6, 12 and 36 months. (DOCX)
