# Supplementary material for: HIV treatment outcomes following antiretroviral therapy initiation and monitoring: A workplace program in Papua, Indonesia
Source: PLoS One. 2019 Feb 25;14(2):e0212432. doi: 10.1371/journal.pone.0212432 (PMC6388914; doi:10.1371/journal.pone.0212432)
Supplement: S1 Table — (DOCX) [file pone.0212432.s001.docx]

S1 Table. Univariate analyses of predictors of HIV treatment outcomes across low, medium, and high groups at 6,12 and 36 months.

|  | **Viral suppression LMIC, VL <1000copies/ml** | | **Poor immune responder, CD4 <200cells/mm3** | | **Immunological failure, CD4 <100cells/mm3** | |
| --- | --- | --- | --- | --- | --- | --- |
| **Variable** | **OR (95% CI)** | **P Value** | **OR (95% CI)** | **P Value** | **OR (95% CI)** | **P Value** |
| **Sex** |  |  |  |  |  |  |
| Male | 1 |  | 1 |  | 1 |  |
| Female | 0.89 (0.42-1.90) | 0.77 | 1.36 (0.65-2.82) | 0.41 | 1.98 (0.84-4.70) | 0.11 |
| **Ethnicity** |  |  |  |  |  |  |
| Papuan7-tribes | **1** |  | **1** |  | **1** |  |
| Papuan non-7-tribes | 1.37 (0.75-2.50) | 0.31 | **0.64 (0.37-1.10)** | **0.1** | 0.61 (0.31-1.20) | 0.15 |
| Non-Papuan | **13.21 (3.30-52.90)** | **<0.001** | **0.28 (0.12-0.68)** | **0.002** | **0.07 (0.01-0.57)** | **0.001** |
| **Age group** |  |  |  |  |  |  |
| 18-29 | 1 |  | 1 |  | 1 |  |
| 30-39 | **2.11 (1.09-4.06)** | **0.02** | 0.61 (0.33-1.12) | 0.11 | **0.43 (0.20-0.94)** | **0.03** |
| 40-49 | 1.52 (0.69-3.33) | 0.29 | 1.18 (0.59-2.37) | 0.64 | 0.74 (0.31-1.76) | 0.49 |
| >50 | - | 0.45 |  |  | - | - |
| **Baseline CD4 group** |  |  |  |  |  |  |
| Low, ≤100 | **1** |  | **1** |  | **1** |  |
| Medium,  >100-350 | **1.78 (0.97-3.28)** | **0.06** | **0.17 (0.09-0.31)** | **<0.001** | **0.15 (0.07-0.34)** | **<0.001** |
| High, >350 | 1.02 (0.48-2.12) | 0.96 | **0.15 (0.06-0.38)** | **<0.001** | **0.33 (0.12-0.85)** | **0.015** |
| **Baseline VL group** |  |  |  |  |  |  |
| Low, <10,000 | 1 |  | 1 |  | 1 |  |
| Medium,  10,000-100,000 | 0.60 (0.12-2.94) | 0.52 | 2.77 (0.33-22.97) | 0.33 | 1.09 (0.12-9.63) | 0.94 |
| High, >100,000 | 0.46 (0.10-2.15) | 0.31 | 6.14 (0.78-48.67) | 0.05 | 2.52 (0.32-19.94) | 0.36 |
| **1 log decrease in VL at 6 months** |  |  |  |  |  |  |
| No | **1** |  |  |  |  |  |
| Yes | **9.58 (3.90-23.58)** | **<0.001** |  |  |  |  |

Note: Significance set at p<0.1.
